# Bioinformatic exploration of the immune related molecular mechanism underlying pulmonary arterial hypertension

**DOI:** 10.1080/21655979.2021.1944720

**Published:** 2021-07-12

**Authors:** Ruina Huang, Xifeng Zheng, Junxian Wang

**Affiliations:** a Department of Cardiology in Affiliated Hospital of Guangdong Medical University, Zhanjiang, Guangdong, People’s Republic of China; b Department of Geriatrics, Affiliated Hospital of Guangdong Medical University, Zhanjiang, Guangdong, People’s Republic of China

**Keywords:** Biomarker, Pulmonary arterial hypertension, CIBERSORT, Immune and inflammation

## Abstract

This study aimed to explore the molecular mechanisms related to immune and hub genes related to pulmonary arterial hypertension (PAH). The differentially expressed genes (DEGs) of GSE15197 were identified as filters with adjusted P value <0.05, and |Log2 fold change|> 1. Biofunctional and pathway enrichment annotation of DEGs indicated that immunity and inflammation may play an important role in the molecular mechanism of PAH. The CIBERSORT algorithm further analyzed the immune cell infiltration characteristics of the PAH and control samples. Subsequently, 16 hub genes were identified from DEGs using the least absolute shrinkage and selection operator (LASSO) algorithm. An immune related gene CX3CR1 was further selected from the intersection results of the 16 hub genes and the top 20 genes with the most adjacent nodes in the protein-protein interaction (PPI) network. GSE113439, GSE48149, and GSE33463 datasets were used to validate and proved CX3CR1 with a remarkable score of AUC to distinguish PAH samples caused by various reasons from the control group.

## Graphical Abstract

**Figure:**
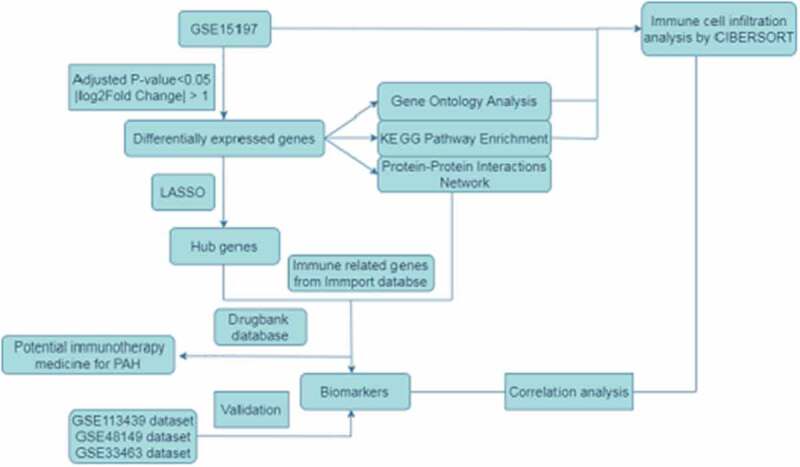

## 1. Introduction

Pulmonary arterial hypertension (PAH) is a clinical and pathophysiological syndrome caused by various heterogeneous diseases (etiology) and pathogeneses associated with pulmonary vascular structure, or functional changes that result in the development of pulmonary vascular resistance and increase in pulmonary artery pressure, subsequently triggering right heart failure or even death. It should be noted that a lowered minimum mean pulmonary artery pressure for diagnosis from 25 to 20 mmHg at rest has been described by the latest revised definition of PAH [1]. Although several medicines and lung transplants had been applied to treatment PAH in clinical during the past decades, the five-year survival rate remains only 50% [2,3].

PAH is considered as an autoimmune inflammatory disease because of the complex changes in cytokines, chemokines, and inflammatory mediators [4]. Marsh LM et al. described the different distributions of 21 types of inflammatory cells in idiopathic pulmonary arterial hypertension (IPAH) patients and normal controls in detail; their study suggested that immune imbalance played an important role in the pathogenesis of IPAH [5]. Since 2015, more than 170 clinical trials focusing on PAH treatment have been registered in the National Library of Medicine clinical trials registry, and approximately 20% of the trials are related to the application of immunotherapy for treating pulmonary hypertension [6]. Additionally, mesenchymal stem cell therapy has also shown potential clinical value in preclinical studies [7,8].

The signaling pathways and regulatory networks underlying the pathogenesis of PAH remain unknown due to the complicated etiologies. In this study, the microarray dataset of GSE15197 was adopted to screen differentially expressed genes (DEGs), exploring the gene ontology (GO), Kyoto Encyclopedia of Genes and Genomes (KEGG) pathway enrichment, protein-protein interaction (PPI) network and further identify the immune related biomarker. We also applied the CIBERSORT algorithm [9], a method to provide an estimation of the abundances of member cell types in a mixed cell population with gene expression data, to analyze the immune cell infiltration characteristics of 22 types of immune cells in PAH and normal samples. The selected biomarker was verified by GSE33463, GSE48149, GSE113439 datasets.

This study aimed to analyze the transcriptional profiles of patients with PAH based on the assessment of immune-related genes and immune cell infiltration characteristics, to explore the mechanism of immune response elicited in the pathogenesis of PAH, and to discover potential diagnostic biomarkers and therapeutic drugs to provide insights for an early diagnosis and for the development of immune-targeted therapy of PAH.

## 2. Materials and methods

### 2.1 Data source and DEGs analyses

The microarray datasets GSE15197 [10], GSE33463 [11], GSE48149 [12] and GSE113439 [13] were sourced from the GEO database. Detailed information about the datasets were provided in Table 1. All data were statistically analyzed and visualized using R software 4.0.3 and relevant packages. DEGs in the GSE15197 dataset were screened with adjusted P-value < 0.05 and |Log2 fold change|> 1 as the empirical analysis cutoff by ‘limma’ package [14].

**Table 1.** Information of microarray dataset from GEO database

Dataset	Tissue	PAH	Control	Platform
GSE15197	Lung tissue	26	13	GPL6480
GSE113439	Lung tissue	15	11	GPL6244
GSE48149	Lung tissue	18	9	GPL16221
GSE33463	Peripheral blood mononuclear cell	80	60	GPL6947

The ‘ClusterProfiler’[15] and ‘org.Hs.eg.db’[16] packages were used to explore the gene ontology (GO) and KEGG pathway enrichment of DEGs with a filter condition as P-value < 0.05. The results were visualized by ‘ggplot2’[17] package. The protein-protein interaction (PPI) network was constructed using STRING (https://string-db.org/,Version: 11.0) [18], an online web tool used to explore functional protein association networks, a filter condition with high confidence, and a minimum required interaction score of more than 0.7.

### 2.2 Exploring the abundance of immune cells in samples

CIBERSORT, a method of exploring 22 types of tissue immune cells based on their gene expression profiles, was adopted to analyze the normalized GSE15197 expression data. A matrix of 22 types of immune cells was obtained, and all samples presented with a P-value < 0.05. The percentage of each immune cell type in the samples was calculated and displayed using a bar plot. The ‘vioplot’ package was used to compare the levels of 22 types of immune cells between the two groups. [19]

### 2.3 Identification of hub-genes and potential immunotherapy medicine prediction

DEGs were screened using the least absolute shrinkage and selection operator (LASSO) algorithm to confirm the hub genes. We were especially interested in detecting potential immunotherapy targets and medicines for PAH, because immunotherapy is a promising field that has not been explored extensively. A dataset with 1793 unique immune-related genes was downloaded from the IMMPORT database^[20]^ (https://www.immport.org/shared/) and used to matched the immune-related hub genes. DrugBank [21] (https://go.drugbank.com/) is a comprehensive pharmaceutical knowledge database, which researchers can use to study drugs which are in trial and/or have been applied clinically. We used this database to screen potential medicines for PAH and direct future studies.

### 2.4 Identification and validation of biomarkers

Potential biomarker of PAH was detected based on the intersections of hub genes with genes that have obvious connections with adjacent nodes in the PPI network. The discriminatory ability of the biomarker was validated using the GSE33463, GSE48149 and GSE113439 datasets and assessed using receiver operator characteristic (ROC) curves.

### 2.5 Correlation analysis between immune cells and biomarkers

Spearman’s rank correlation analysis was used to analyze the relationship between 22 types of immune cells and the biomarker by ‘ggstatsplot’[22] and ‘ggplot2’ package. P-value < 0.05 was considered statistically significant.

## 3. Results

Our study indicated that, according to GO and KEGG enrichment analysis, inflammation and immune response played important roles in the pathogenesis of pulmonary hypertension. Through further analysis of immune cell infiltration, mast cells, B cells, monocytes, and neutrophils were with statistical difference (P value <0.05) and should be explored in detail. As an immune-related hub gene, CX3CR1 presents with a promising ability to be used to distinguish between patients with pulmonary hypertension caused by various reasons and those in the control group and can be considered a potential biomarker of pulmonary hypertension.

### 3.1 DEGs between PAH and control samples and the biofunctional analysis

**Figure 1.**
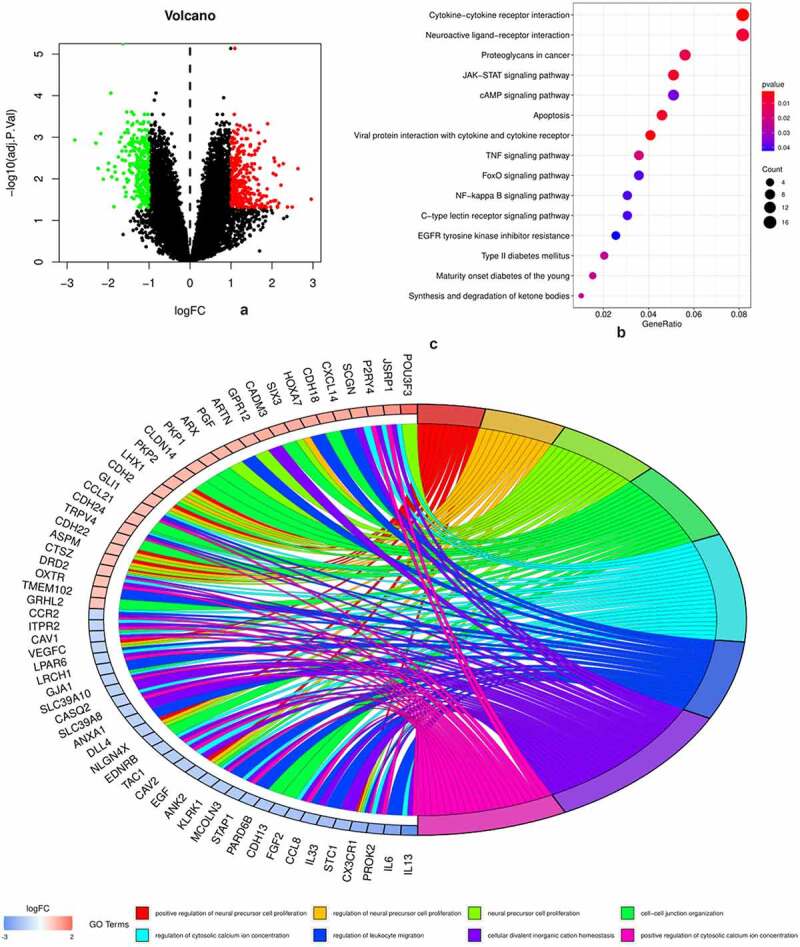
DEGs and biofunctional analysis

**Figure 2.**
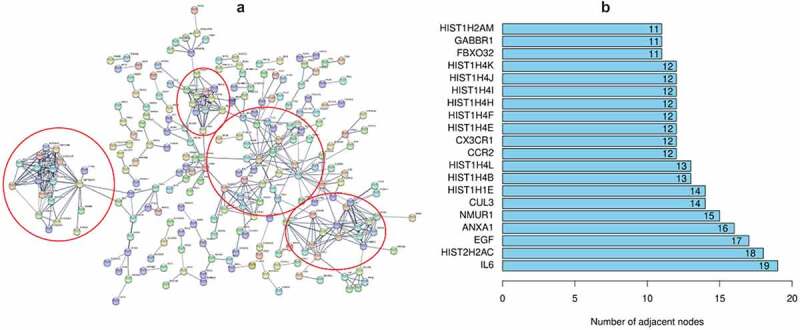
The PPI network of DEGs and the top 20 genes with most adjacent nodes

A volcano plot (Figure 1(a)) was used to visualize the results of 587 DEGs, of which 321 were upregulated and 266 were downregulated. The results of KEGG pathway enrichment and gene ontology (GO) analysis are shown in Figure 1(b,c). It is obvious that inflammation and immune-related pathways and biological regulatory functions play a dominant role in the enrichment results of the DEGs of PAH. The PPI network of DEGs and the top 20 genes with the most adjacent nodes are shown in Figure 2.

Figure 1(a): Volcano plot of 587 DEGs, of which 321 were upregulated (red) and 266 were downregulated (green). Figure 1(b): Top 20 KEGG pathways enriched of the DEGs. Figure 1(c): Top eight gene ontology (GO) enrichments of the DEGs.

The Figure 2(a) displays the PPI network of DEGs. Each small circle represents a gene. It is obvious in the picture that some genes are highly concentrated as a module and with interaction connect to other genes. The Figure 2(b) listed the top 20 genes in the PPI network with most adjacent nodes.

### 3.2 Immune cell infiltration of the samples

The result of CIBERSORT arithmetic analysis show the difference in abundance of 22 immune cells in the PAH and control samples. B cell naïve, monocytes, resting mast cells, eosinophils, and neutrophils were expressed at higher levels in control samples, while B cell memory and mast cell activation were significantly higher in the PAH group (*P* < 0.05). The matrix of result was listed in supplement file1.

**Figure 3.**
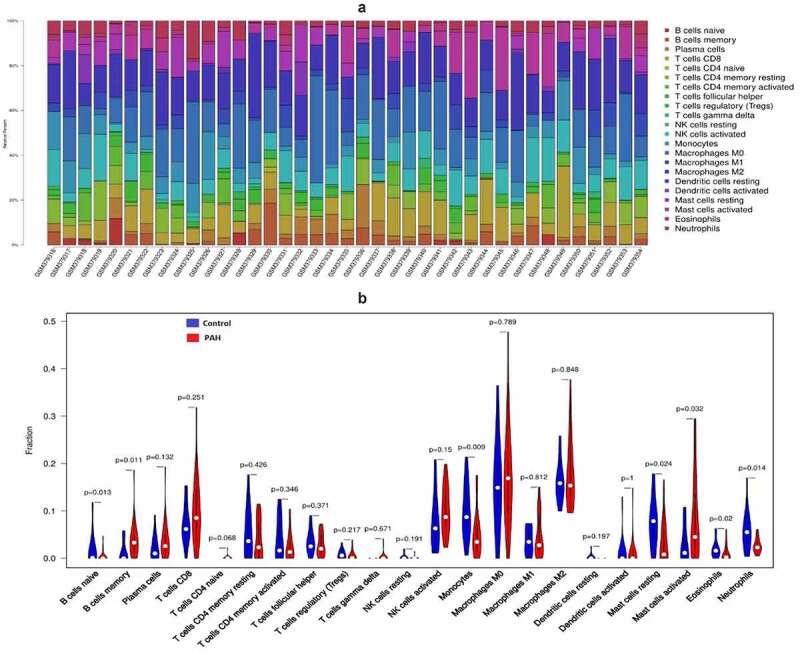
Immune cell infiltration in PAH and control samples

The Figure 3(a) displays the proportions of immune cell subsets in the PAH and control samples. The Figure 3(b) displays statistically significant differences in immune cell subsets.

### 3.3 Hub genes identification and potential immunotherapy medicine prediction

**Figure 4.**
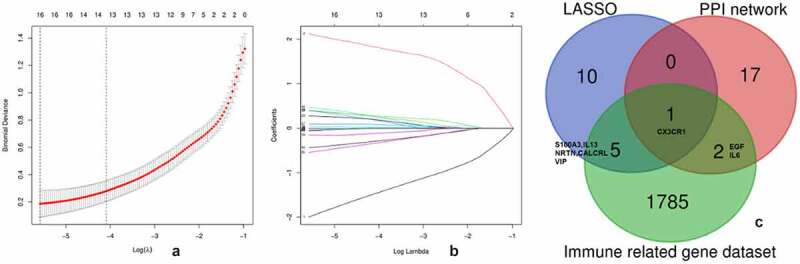
Hub genes identified by LASSO

The LASSO algorithm was used to screen hub genes from the DEGs; as a result, 16 hub genes (SIAE, AKR7A2P1, NAT14, NRTN, SLC45A4, ADAD2, S100A3, LOC283481, CALCRL, IL13, VIP, GRHL2, SCG5, ABI3BP, CX3CR1, IFI44L) were identified (Figure 4(a) and Figure 4(b)). Three genes among the top 20 genes with most adjacent nodes in PPI network (CX3CR1, IL6, EGF) and six genes (S100A3, IL13, NRTN, CALCRL, VIP) among the hub genes by LASSO were matched with the immune related gene original from IMMPORT database (Figure 4(c)). The nine immune related genes were further investigated in the DrugBank database to predict potential medicines for PAH treatment, and the results are listed in Table 2.

**Table 2.** The potential medicine of immune related genes predicted in drugBank

Genes	Protein name	Drugs	State	DrugBank ID
IL13 EGF CALCRL IL6	Interleukin-13 Epidermal growth factor Calcitonin gene-related peptide type 1 receptor Interleukin-6	Lebrikizumab Erlotinib Dacomitinib Ubrogepant Rimegepant Erenumab Siltuximab	Investigational Approved Approved Approved Approved Approved Approved	DB11914 DB00530 DB11963 DB15328 DB12457 DB14039 DB09036

The Figure 4(a) displays the optimal lambda (0.003) selection in the LASSO regression with 16 hub genes. The Figure 4(b) displays the coefficient profiles of the 16 hub genes. The Figure 4(c) displays the 3 immune related gene in PPI network and 6 immune related gene among the 16 hub genes selected by LASSO.

### 3.4 Identification and validation of PAH biomarker

**Figure 5.**
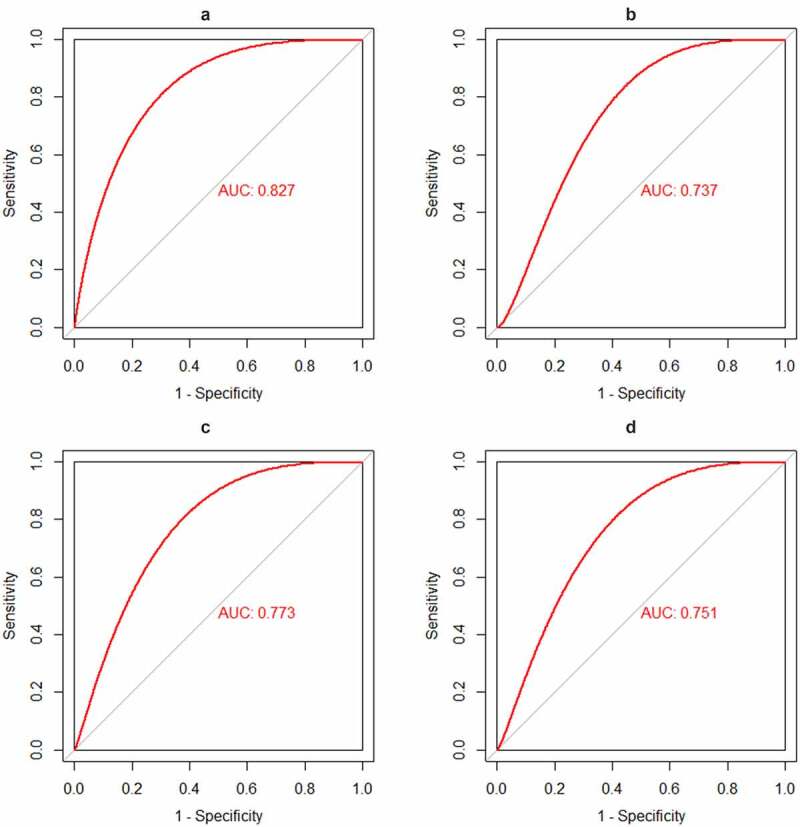
Identification and validation the biomarkers of PAH

**Figure 6.**
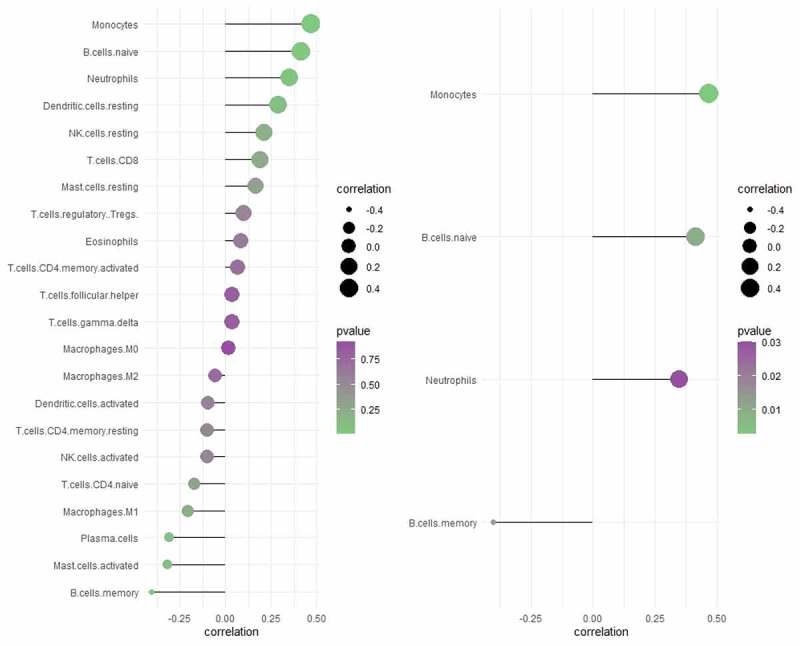
Correlation analysis between CX3CR1 with 22 immune cells

CX3CR1 was the only immune related gene considered as potential biomarker for intersecting with 16 hub genes and the top 20 genes with the most adjacent nodes in the PPI network. The discriminatory ability of CX3CR1 was validated using the GSE33463, GSE48149 and GSE113439 datasets. As shown in Figure 5(a), CX3CR1 showed promising discriminatory ability of PAH in GSE15197 (AUC = 0.827), with a slight but acceptable decrease in GSE113439 (Figure 5(b), AUC = 0.737), GSE48149 (Figure 5(c), AUC = 0.773) and GSE33463 (Figure 5(d), AUC = 0.751). Correlation analysis showed that CX3CR1 was mainly positively correlated with monocytes, B cells and neutrophils (Figure 6) with P-value < 0.05.

The receiver operating characteristic (ROC) curve of the discrimination ability of CX3CR1. Figure 5(a): AUC of CX3CR1 in GSE15197, Figure 5(b): AUC of CX3CR1 in GSE113439, Figure 5(c): AUC of CX3CR1 in GSE48149, Figure 5(d): AUC of CX3CR1 in GSE33463.

The left image shows correlation analysis between CX3CR1 with 22 types of immune cells. The right image shows the immune cells with the strongest correlation with CX3CR1 and P-value< 0.05. The size of the dots represents the strength of correlation between gene biomarkers and immune cells. The color of the dots represents the P-value.

## 4. Discussion

PAH is a rare but life-threatening disease that can be categorized into five types according to the different dominant etiologies [23]. Unfortunately, the treatment of PAH remains unsatisfactory, because of these multifactorial etiologies that result in a complex and uncertain pathophysiological state. In the current literature, genetic and/or gene mutations, inflammation, immunity, endothelial dysfunction, and abnormal vascular wall cell proliferation are closely related to the development of PAH [24,25]. We systematically analyzed the transcriptional map of PAH and explored the potential treatment medicine using bioinformatics to reveal the underlying molecular mechanisms and pathological processes, which may contribute to the prevention and treatment of the disease.

GO and KEGG pathway enrichment analyses revealed a strong involvement of inflammation and immune related process and pathways in PAH, indicating that they may be essential to the pathological processes of PAH. Based on these instructions, we explored the characteristics of 22 types of immune cell infiltration between PAH and normal samples, using the CIBERSORT algorithm. Naïve B cells, monocytes, resting mast cells, and neutrophils were expressed at higher levels in control samples (P-value < 0.05), while memory B cell and mast cell activation were significantly higher in the PAH group (P value < 0.05). Thus, the immune cells mentioned above, especially mast cells and B cells, may be closely related to the progression of PAH, a hypothesis which is supported by abundant evidences [26,27].

Mast cells were observed to be enriched in the pulmonary vascular tissue of PAH, and even appeared in the adjacent tissue of pulmonary perivascular cytopathy in the early stage. In a left heart failure-related pulmonary hypertension rat model treated with ketotifen (mast cell stabilizer) and a WS/WS rat model with mast cell deficiency (due to the mutation of mast cell growth factor receptor c-kit), the symptoms and vascular remodeling of pulmonary hypertension were significantly reduced. Both strategies were also effective and achieved similar results in monocrotaline-induced pulmonary arterial hypertension, suggesting that the role of mast cells extends to PAH caused by different pathogenesis [28–30].

Similar to mast cells, B cells have also been directly observed infiltrating pulmonary perivascular tissue and show strong activation in the peripheral blood of PAH patients [31].

Ulrich et al. reported that B cells have remarkably upregulated gene expression in the peripheral blood of PAH patients compared to healthy controls. The differentially expressed genes are mainly involved in vasomotor regulation, angiogenesis, and cell proliferation [32].

Antibodies originate from B cells, which is the basis of the humoral immune response. Antibodies against pulmonary endothelial cells and fibroblasts have been detected in PAH tissue, and pathogenic auto-antibodies targeting endothelial cells are capable of inducing vascular endothelial apoptosis, and may initiate the development of PAH, which suggests that B cells may promote the pathological process of PAH [33–36].

Immune cells and immune related genes are the focus of this study. According to the data set of 1793 immune related genes provided by IMMPORT database, we further selected 9 immune related genes to explore potential therapeutic drugs by Drugbank database.

Lebrikizumab is a humanized monoclonal antibody against interleukin-13 (IL-13) which has been used in trials studying the treatment of allergic asthma, atopic dermatitis, and idiopathic pulmonary fibrosis (IPF) by inhibiting the IL-13 driven Th2 inflammatory response [37–39]. Erlotinib and dacomitinib are inhibitors of the epidermal growth factor receptor (EGFR) tyrosine kinase and are used to treat non-small cell lung cancer, pancreatic cancer, and several other cancer types. Accumulating evidence suggests that EGFR is closely associated with the molecular mechanism of PAH, and epidermal growth factor inhibitors such as erlotinib and dacomitinib have been used to treat PAH in animal models [40–42].Interleukin-6 (IL-6) is a pleiotropic cytokine with a wide range of biological activities such as immune regulation, hematopoiesis, inflammation, and oncogenesis. By forming a high-affinity complex with human interleukin-6 (IL-6) to prevent IL-6 from binding to soluble and membrane-bound IL-6 receptors, stuximab is used to treat Castleman’s disease, a type of lymphoproliferative disease [43]. Recent studies have verified that it can improve pulmonary ventilation function and reduce mortality in patients with severe COVID-19 [44,45]. It should be noted that the IL-6 receptor antagonist, tocilizumab, has been used in clinical trials to treat pulmonary arterial hypertension [46].

Through drug prediction of hub immune-related genes, it can be inferred that immune-targeted drugs for different primary diseases demonstrate extensive research applications in the treatment of PAH. This is because there is an important immune mechanism underlying PAH. Exploration of the common immune pathway of PAH caused by different causes is of certain significance for the treatment of PAH.

CX3CR1 was considered as potential biomarker for the intersection result of the 16 hub genes and the top 20 genes with the most adjacent nodes in the PPI network. The discrimination ability of CX3CR1 was promising in both lung tissue and peripheral blood testing. CX3CR1 is the receptor for fractalkine and is regarded as a mediator of vascular and tissue damage in PAH. Jonathan Florentin et al. demonstrated that CX3CR1 played an important mediating role in the mobilization of myeloid cells from the bone marrow to blood and then to the lungs in patients with PAH [47]. An increase in chemokine levels in lung tissues and the expression of chemokine receptors in blood monocytes initiate the migration of inflammatory monocytes to lung tissues. These monocytes differentiate into perivascular stromal macrophages, secrete proinflammatory cytokines, and promote vascular remodeling. Similarly, CX3CR1 also regulates neutrophil migration [48]. From the perspective of CX3CR1 deficiency, some studies have confirmed that hypoxia-induced PAH can be prevented by reducing monocyte recruitment, macrophage polarization, and pulmonary artery smooth muscle cell proliferation [49]. These studies indicate that CX3CR1 is an important regulatory target in the mechanism of inflammatory cell migration to lung tissues in patients with PAH. The results of the correlation analysis showed that CX3CR1 had the medium positive correlation with monocytes and neutrophils among 22 types of immune cells, which is consistent with the known studies. Currently, there is no medicine targeting CX3CR1, and a deep research about CX3CR1 and its ligand may be useful to direct the development of medicine development.

## 5. Conclusions and limitations

In this study, we systematically analyzed the transcriptional profiles of PAH, explored the role of immune cells in the pathogenesis of PAH, identified and verified a promising biomarker, CX3CR1. However, further in-depth studies on inflammation and immunity and validation in a larger population are essential to fully reveal the pathological processes and molecular mechanisms of PAH and to develop target medicines.

## Supplementary Material

Supplemental Material

## Data Availability

The datasets used and/or analyzed during the current study are available from the Gene Expression Omnibus (GEO) database.

## Highlights:

1. Inflammation and immune response play important roles in the pathogenesis of pulmonary arterial hypertension.

2. CX3CR1 is an important regulatory target and potential biomarker in pulmonary arterial hypertension.

3. Immune related targeted drugs may be effective in the treatment of pulmonary arterial hypertension.
